# Improving fit to work assessments for rail safety workers by exploring work limitations

**DOI:** 10.1007/s00420-016-1117-7

**Published:** 2016-02-11

**Authors:** J. S. Boschman, C. T. J. Hulshof, M. H. W. Frings-Dresen, J. K. Sluiter

**Affiliations:** Academic Medical Center, Coronel Institute of Occupational Health, University of Amsterdam, PO Box 22660, 1100 DE Amsterdam, The Netherlands

**Keywords:** Occupational health, Occupational safety, Transportation, Health status, Work fitness

## Abstract

**Purpose:**

We aim to provide evidence for improving fit to work assessments for rail safety workers and raised the question whether adding an assessment of work limitations is useful. Therefore, we assessed differences in the proportions of perceived work limitations and reported health complaints and whether older age or having health complaints are risk factors for having work limitations.

**Methods:**

Job requirements for rail safety workers are ‘vigilance and clear judgment’, ‘good communication abilities’, ‘sufficient eye sight’ and ‘task-required physical abilities’. We invited 1000 workers to fill in a questionnaire about perceived work limitations and health problems related to their job requirements. Proportions of the two were compared by using the McNemar test. Associations were analyzed by using univariate logistic regression.

**Results:**

Among 484 rail safety workers, we found statistically significant differences between the proportions of reported health complaints (2–26 %) and work limitations (10–32 %). No significant associations were found between older age and work limitations, except for workers in the age group 40–50 years regarding physical abilities. This was not found for the age group over 50 years. For each age category, workers reporting health complaints related to ‘vigilance and clear judgment’ and ‘sufficient physical abilities’ had a statistically significant increased risk for reporting work limitations as well (ORs 2.4–17.9).

**Conclusions:**

Our results indicate that fit to work assessments should include both health complaints and work limitations. Our results do not substantiate the assumption that workers over 40 years of age are at increased risk for work limitations in general.

## Introduction

In a number of occupations, workers are required to undergo fit to work assessments to safeguard their personal health and safety as well as to reduce risks to other workers or other persons. Examples are: commercial drivers, fire fighters, pilots, crane operators and rail safety workers. The workers are examined for being fit to perform routine and emergency duties and to verify that they are not suffering from any medical condition that would render them unfit for service or would endanger the health of other persons (International Labour Office 2013). Obligatory fit to work assessments are any medical examinations of an employee during the employment based on a statutory duty or obligation under a collective agreement to which an employee is required to subject to (NVAB 2007).

Routine medical inspections or health examinations in occupational groups are not new: in 1941, Morris argued that regular health examinations would be a major advance in public health (Morris and Glasg 1941). Traditionally, obligatory fit to work assessments are designed much like periodic health examinations offered to workers, in which mainly the health status of the worker is examined (Storer et al. 2014; Thiese et al. 2015). This contrasts sharply with the idea that medical fitness should always be judged in relation to the work, as many medical conditions have minimal implications for work (Palmer and Brown 2013). Additionally, an assessment of the medical fitness for work is not a goal in itself: it must be maintained by timely interventions to restore health, reduce work limitations and sustain work ability. Criticism on fit to work assessments for workers concerns in particular the lack of evidence of benefit of the examinations (Mahmud et al. 2010; Ren et al. 1994). The question arises whether the assessment of health complaints and diseases is sufficient to detect workers with possible work limitations.

The assessment of job-specific work performance and work limitations in order to improve the quality of workers’ health surveillance programs has been used, for example, for fire fighters (Plat et al. 2010a, b), hospital nurses (Gartner et al. 2012) and construction workers (Boschman et al. 2013). It is assumed that health complaints could result in impaired work functioning, even though the worker might not always be aware of the presence of health problems and their consequences.

The occupation of rail safety worker is one in which the workers are obliged to undergo a fit to work assessment (NTC Australia 2012; Rail Safety and Standards Board Limited 2011). In the Netherlands, workers aged below 40 years are assessed every 4 years, once in every 2 years when they are between 40 and 50 years of age, and annually when they are 50 years or older. The time intervals of the medical exams differ, depending on national or local legislation (NTC Australia 2012). It is unknown whether there is a scientific basis for the age dependency of the frequency of the fit to work assessments for rail safety workers.

In the Netherlands, rail safety workers are assigned to safeguard the rail maintenance workers for all types of work that involves railroad. As any infrastructure, railways are periodically inspected and subject of maintenance in order to minimize effect of infrastructure failures that can disrupt freight revenue operations and passenger services. Rail workers perform the general day-to-day maintenance of the railway such as looking after tracks, signals and power supplies, but also engineering work and larger-scale projects such as track replacement.

In the Netherlands, six distinct roles that rail safety workers perform during railroad maintenance activities can be distinguished: workplace safety leader (the worker oversees the project and is responsible for the safety of all workers at the various worksites during a project), local rail safety leader (the worker oversees one local worksite and is responsible for the workers at that worksite), safety watchman (warns the workers for approaching trains at a worksite when the railroad is in use), border safety man (warns the workers when they are too close to a railroad that is in use), escort of railroad equipment (safeguards transportation of railroad equipment and works in close harmony with the operator of the equipment), operator of railroad equipment (operates the railroad equipment). Some of the tasks can be combined as the worker may perform them one after the other, for example: escorting railroad equipment to the worksite, and after arriving, switching to the task of border safety man. When the worker performs a rail safety task, he or she does not carry out other rail maintenance work (Boschman et al. 2014a).

The aim of this exploratory cross-sectional questionnaire study is to provide evidence for improving the content of obligatory fit to work assessments for rail safety workers by adding an assessment of work limitations to questions about health complaints. The research questions are as follows:

1. What is the difference in the proportions of perceived work limitations and reported health complaints?
2. What is the association between older age (40–50 years or over 50 years of age) and self-perceived work limitations among rail safety workers?
3. What is the association between having health complaints and perceiving work limitations for each age category (<40 years, 40–50 years or over 50 years of age)?

## Methods

### Sample size and procedure

A priori, we aimed at detecting a difference of 10 % between the prevalence of health complaints and work limitations with a precision of 5 % and power of 90 %. We expected the proportion of discordant pairs to be around 30 %. This leads to an estimated sample size of at least 412 (Elashoff 2011). Based on previous questionnaire surveys among similar populations, a 40 % (Boschman et al. 2012) to 70 % (Ganasegeran et al. 2014) response rate was expected. Therefore, approximately 1000 rail safety workers were approached and invited to participate in the study. We asked the four largest railroad construction contractors and 69 smaller workplace security companies in the Netherlands to inform their rail safety workers about the study. The workers were invited to participate voluntarily and to complete an online questionnaire. The whole sector was represented in this sample. In the Netherlands, the definition of ‘rail safety worker’ does not include railway maintenance workers.

A cross-sectional survey was conducted in November 2013. In close consultation with the employers, we chose the most appropriate strategy to inform the workers: by email or by a letter. One employer facilitated the workers by having computers with internet access available at the workplace for them. We pilot-tested and used similar questionnaires among construction workers (Boschman et al. 2012). The study complied with the institutional ethical approval requirements and was performed in accordance with the ethical standards as laid down in the 1964 Declaration of Helsinki and its later amendments. The workers were informed about the aims of the study and asked to give their written informed consent. Informed consent was obtained from all individual participants included in the study. Completing the questionnaire took approximately 15 min. One reminder was sent to all participants after 2 weeks.

### Questionnaire

The online questionnaire comprised the following parts: informed consent, personal characteristics (gender, age), job characteristics (current rail safety tasks, other rail maintenance tasks, number of hours worked during the previous week, number of years employed in rail safety), job-relevant health complaints, work limitations, and safety issues and accidents. In a preparatory phase of this study, we conducted group interviews with 3–5 workers on each specific rail safety task. During each interview, the following topics were discussed with the workers: job tasks and demands, type of activities and the duration, intensity and frequency of these activities, the variation in tasks and activities on regular work days and differences between work days, the perceived load of the tasks, health complaints that interfere with safe task performance according to the workers own experience, and potentially dangerous situations that might occur (Boschman et al. 2014a).

Based on the group interviews and in accordance with Dutch law (Ministry of Social Affairs and Employment 1998), we considered the following job requirements of importance in these fit to work assessments: vigilance and clear judgment, good communication abilities, sufficient eyesight, and task-required physical abilities. Based on these job requirements, we determined to which health complaints a fit to work assessment should be directed, based on the rationale that those health complaints could influence the workers’ ability to meet the job requirements and fulfill the criteria for screening (Andermann et al. 2008; Sluiter et al. 2013).

Next, for each individual rail safety task we defined relevant work limitations. Workers were asked only about limitations relevant for the rail safety task or tasks they performed. The items were scored on a 4-point scale (0 = never, 1 = sometimes, 2 = frequently, 3 = always). Examples of our self-formulated questions are: ‘Do you currently experience problems/difficulties in remaining concentrated when on duty?’ and ‘Do you currently experience problems/difficulties in communicating with the railway traffic controller?’. The content of the questionnaire regarding health and work limitations is listed in Table 1.

**Table 1:** Content of the questionnaire for rail safety workers: health complaints and work limitations relevant for the workers’ ability to meet their job requirements

Job requirement	Health	Work limitations (specific for the individual rail safety tasks)
Vigilance and clear judgment	*Chronic or previous health conditions* Fainting/diminution of consciousness; Diabetes mellitus, thyroid gland disorder; Sleep apnea; Epilepsy; Migraine; High blood pressure, cardiac arrhythmia, heart condition, heart attack; Psychosis, schizophrenia; Alcohol or substance abuse, addiction. *Present health issues* Increased need for recovery after work Symptoms of: Depression; Distress; Anxiety; Post-traumatic stress disorder; Sleepiness.	*Limitations regarding the ability to* Remain concentrated when on duty; Make decisions.
Sufficient communication abilities	*Chronic health conditions* Noise-induced hearing loss/deafness; Disorders that affect speech.	*Limitations regarding the ability to* Communicate, also by phone/walkie-talkie; Communicate with the railway traffic controller; Instruct/warn the workers; Hear warning signs.
Sufficient eye sight	*Chronic health conditions* Color blindness; Night blindness; Cataract or retinitis.	*Limitations regarding the ability to* Read work and safety instructions and diagrams; Read from telephone/tablet screen; Read indicators in the vehicle/machine; See the railroad workers (by day and night); See the railroad workers and nearby equipment (by day and night); See the train at 30 s sight; Distinguish the train from its surroundings.
Sufficient physical abilities	*Chronic health conditions* Arthrosis, or rheumatism; Hernia, slipped disk. *Present health issues* Musculoskeletal complaints.	*Limitations regarding the ability to* Operate telephone/tablet; Walk, also in the ballast; Getting in and out of the car; Getting in and out of the vehicle/machine; Alternately standing and walking; Prolonged standing; Continuously looking from left to right and vice versa; Sitting in the vehicle/machine; Carrying safety equipment to the workplace.

Workers were asked to indicate whether or not they were currently suffering, or had ever suffered from the following chronic or other health conditions (yes/no): fainting/unconsciousness; diabetes mellitus/thyroid gland disorder; sleep apnea; epilepsy; migraine; high blood pressure/cardiac arrhythmia/heart condition/heart attack; psychosis, schizophrenia; alcohol or substance abuse, addiction; noise-induced hearing loss/deafness; disorders that affect speech; color blindness; night blindness; cataract or retinitis; arthrosis, or rheumatism; hernia, slipped disk; current musculoskeletal complaints.

Need for recovery after work (van Veldhoven and Broersen 2003) was included as this concept bridges the stage between normal work-related effort and serious long-term work-related fatigue syndromes, such as burnout (Sluiter et al. 2003). The need for recovery scale (Cronbach’s alpha: 0.78) has been found to be an adequate measure for early symptoms of fatigue from work, for use in scientific research (van Veldhoven and Broersen 2003). The eleven items are answered with either a ‘yes’ or ‘no.’ Workers answering more than six items positively are likely to have an increased risk for psychological complaints (Broersen et al. 2004).

Four scales rated common mental health disorders by measuring self-reported symptoms indicative for distress, anxiety, depression and PTSD. Distress was measured with the distress screener (Cronbach’s alpha: 0.83) developed by Braam et al. (2009). The distress screener was found to be a valid tool for early identification of distress in workers (Braam et al. 2009). The items are answered on a 3-point scale, ‘no’ (0), ‘sometimes’ (1) or ‘regularly or often’ (2), indicating the respondent’s level of agreement with the question. A total score was constructed by the sum of the answers on the three items. The cutoff point that distinguishes between ‘screened positive’ and ‘screened negative’ was set at a score of 4 or higher (Braam et al. 2009). A positive score means that the person involved is scored as distressed according to the distress screener.

Symptoms indicative of depression and anxiety were detected with the corresponding subscales of the Brief Symptom Inventory (BSI) (de Beurs 2006). Each subscale has six items with a 5-point response scale (0 = not at all, 4 = extremely). Cronbach’s alphas are 0.87 for both scales (de Beurs 2006). For both subscales, mean scores of ≥0.42 are used for case identification, with a sensitivity of 0.86 and a specificity of 0.66 for depression and a sensitivity of 0.83 and a specificity of 0.62 for anxiety (de Beurs and Zitman 2006).

The Dutch version of the Impact of Event Scale (IES) (Brom and Kleber 1985) was used to detect workers with symptoms of PTSD (Cronbach’s alpha: 0.94). The 22 items were scored on a 4-point scale (0 = never, 1 = rarely, 3 = sometimes, 5 = frequently), and a total score was constructed by the sum of the answers on all items. The cutoff value of 26 was used to distinguish the workers with possible PTSD (Horowitz et al. 1979; van der Ploeg et al. 2004).

### Analysis

Two new dichotomous variables were created for each of the four job requirements: one or more health complaints (i.e., screened positive on health complaints) versus no health complaints (i.e., not screened positive on health complaints) and one or more work limitations (i.e., screened positive on work limitations) versus no work limitations (i.e., not screened positive on work limitations). The prevalence of health complaints and work limitations for each job requirement were calculated and confidence intervals (CI) were calculated using the Wald method. Differences between the proportion of workers screened positive on health complaints and screened positive on work limitations were tested using a McNemar test.

As the frequency of the obliged fit to work assessments depends on the workers’ age (once in every 4 years when the worker is under the age of 40, every 2 years when the worker is between 40 and 50 years old, and annually when the worker is 50 years and older), we have chosen to describe the relation between work limitations and age using a matching categorical age variable instead of a continuous outcome. The associations between work limitations and age (with the youngest category as reference category) were analyzed using univariate logistic regression. Next, for each age group we analyzed the association between work limitations and health complaints to give insight into the effect of health complaints.

Statistical significance was set at an alpha level of 0.05. The IBM SPSS Statistics 20.0 software was used to analyze the data.

## Results

In total, 48 % (*n* = 484) of the questionnaires were completed and eligible for analysis. All respondents were active in their current occupation during the previous 6 months. They performed one or more of the six rail safety tasks (workplace safety leader, local rail safety leader, safety watchman, border guardsman, escort of railroad equipment, operator of railroad equipment). More than one-third of the workers (35 %) performed more than one rail safety task, for example workplace safety leader and local rail safety leader or safety watchman and border safety man. Almost all respondents were male (99 %). A description of the tasks and age of the respondents is presented in Table 2. A total of 24 respondents did not fill in their age.

**Table 2:** Characteristics of the respondents (*n* = 484) in a survey among rail safety workers (November 2013)

Rail safety task	Number of respondents	Percentage (%)	Age (years) Mean (SD; min–max)
Workplace safety	290	60	47 (8.6; 25–63)
Local railroad safety	330	68	47 (8.8; 23–64)
Safety watchman	334	69	48 (8.6; 25–64)
Border safety man	389	80	47 (8.6; 25–64)
Operator of railroad equipment	144	30	47 (8.2; 26–63)
Escort of railroad equipment	190	39	49 (8.7; 26–63)

We found that among this active population of rail safety workers a substantial proportion of workers (27–56 %) screened positive on either self-reported health complaints (2–26 %), self-reported work limitations (10–32 %), or both (1–20 %). Details can be found in Table 3. Regarding the job requirement ‘vigilance and clear judgment,’ significantly more workers were found with health complaints possibly affecting their vigilance and decision making (26 %) compared to workers reporting difficulties with their concentration and decision making (10 %) (*P* < 0.001). For all other job requirements, we found a significantly higher proportion of workers (23–32 %) who reported having work limitations in comparison with the proportion of workers who reported having health complaints relevant to that specific job requirement (all *P*’s < 0.001) (Table 4).

**Table 3:** Prevalence of health complaints among rail safety workers participating in a survey (*n* = 484)

Variable	Relative frequency	Percentage (%)
*Current health complaints*		
Increased need for recovery after work	72/448	16
Depression	93/423	22
Distress	91/423	22
Anxiety	34/423	8
PTSD	26/415	6
Sleepiness	40/411	10
Musculoskeletal complaints	62/436	14
*Chronic or previous health conditions*		
Fainting/diminution of consciousness	2/409	0.5
Sleep apnea, epilepsy, migraine	30/409	7
High blood pressure, cardiac arrhythmia, heart condition, heart attack	72/409	18
Psychosis, schizophrenia	3/409	1
Alcohol or substance abuse, addiction.	4/409	1
Color blindness, night blindness, cataract or retinitis	11/409	3
Diabetes mellitus, thyroid gland disorder	23/409	6
Noise-induced hearing loss/deafness	52/409	13
Disorders that affect speech	4/409	1
Arthrosis, or rheumatism, hernia, slipped disk	72/409	18

**Table 4:** Proportion of workers screened positive on health complaints and/or work limitations that could affect the workers’ ability to meet their job requirements (*n* = 484)

Job requirement	Screened positive on health complaints, but not on work limitations	Screened positive on work limitations, but not on health complaints	Screened positive on both health complaints and work limitations	Screened negative on both health complaints and work limitation
Vigilance and clear judgment* *n* (%)	126 (26)	49 (10)	95 (20)	214 (44)
Communication abilities* *n* (%)	31 (6)	112 (23)	23 (5)	318 (66)
Eyesight* *n* (%)	9 (2)	119 (25)	2 (1)	354 (73)
Physical abilities* *n* (%)	19 (4)	157 (32)	87 (18)	221 (46)

* P < 0.001, indicating the statistical significance of differences between the proportion of workers screened positive on health complaints and work limitations (McNemar test)

We found no statistically significant association between work limitations and the three different age groups, except for limitations regarding physical abilities. Workers in the age group 40–50 years are at increased risk (OR 1.7, 95 % CI 1.0–2.9) for having problems or difficulties regarding physical requirements compared to their younger colleagues (Table 5).

**Table 5:** Age-related odds ratio (OR) and 95 % confidence interval (95 % CI) for work limitations (*n* = 460)

Work limitations related to	OR	95 % CI
Vigilance and clear judgment		
Age <40 years (*n* = 92)	Reference	
Age 40–50 years (*n* = 152)	0.99	0.6–1.7^NS^
Age 50 years and older (*n* = 216)	0.71	0.4–1.2^NS^
Communication abilities		
Age <40 years (*n* = 92)	Reference	
Age 40–50 years (*n* = 152)	1.2	0.7–2.1^NS^
Age 50 years and older (*n* = 216)	0.98	0.6–1.7^NS^
Eyesight		
Age <40 years (*n* = 92)	Reference	
Age 40–50 years (*n* = 152)	1.2	0.6–2.2^NS^
Age 50 years and older (*n* = 216)	1.5	0.9–2.8^NS^
Physical abilities		
Age <40 years (*n* = 92)	Reference	
Age 40–50 years (*n* = 152)	1.7	1.0–2.9*
Age 50 years and older (*n* = 216)	1.4	0.9–2.3^NS^

*NS* not statistically significant

* *P* < 0.05, indicating the statistical significance of the association

With respect to the job requirements ‘vigilance and clear judgment’ and ‘sufficient physical abilities,’ we found for each age category that workers reporting health complaints had a statistically significant increased risk for reporting work limitations as well (ORs ranging from 2.4 to 17.9). For the job requirements such as ‘sufficient communication abilities’ and ‘sufficient eyesight,’ this general association could not be confirmed (Table 6).

**Table 6:** Associations between having health complaints and perceiving work limitations for each age category (*n* = 460)

Work limitations related to	OR	95 % CI
Vigilance and clear judgment		
Age <40 years (*n* = 92)	4.3	1.7–11**
Age 40–50 years (*n* = 152)	3.8	1.9–7.7***
Age 50 years and older (*n* = 216)	2.4	1.3–4.4**
Communication abilities		
Age <40 years (*n* = 92)	4.1	0.8–20^NS^
Age 40–50 years (*n* = 152)	1.0	0.2–4.0^NS^
Age 50 years and older (*n* = 216)	2.4	1.1–5.1*
Eyesight		
Age <40 years (*n* = 92)	n.a.	
Age 40–50 years (*n* = 152)	n.a.	
Age 50 years and older (*n* = 216)	0.6	0.07–5.6^NS^
Physical abilities		
Age <40 years (*n* = 92)	17.9	2.2–147.2**
Age 40–50 years (*n* = 152)	4.7	2.0–11.0***
Age 50 years and older (*n* = 216)	5.7	2.7–12.2***

n.a. Not applicable, final model solution could not be found as there were too few workers with health complaints and/or limitations

*NS* not statistically significant

* *P* < 0.05; ** *P* < 0.005; *** *P* < 0.001, indicating the statistical significance of the association

## Discussion

In the present cross-sectional study, we found that the proportion of rail safety workers who perceive work limitations is statistically significantly different from reported health complaints. Older rail safety workers (40–50 years or over 50 years of age) do not appear to be at increased risk for work limitations in general, compared to their younger colleagues. Only regarding physical abilities we found that workers in the age group 40–50 years are at increased risk for limitations. This was not the case for the age group over 50 years.

Based on these results, we recommend that an assessment of the fitness for duty of rail safety workers by means of fit to work assessments should include an assessment of work limitations in addition to the assessment of health complaints. Additionally, we found that across all age categories, workers who reported health complaints related to vigilance and clear judgment and physical abilities had a statistically significant increased risk for perceiving work limitations on these job requirements. Older age in itself was not a risk factor for work limitations in general. Therefore, when the aim is to screen for workers at risk for work limitations, there seems to be no basis for an age-specific approach.

Our findings also showed that among an active population of rail safety workers, up to 56 % of the workers screened positive on either self-reported health complaints, self-reported work limitations, or both. In the case of vigilance and clear judgment, an additional 10 % of the workers are screened positive on top of those who are screened positive based on health complaints. An additional 10 % with respect to vigilance and clear judgment seems little compared to the other topics, such as physical abilities. However, as only questions were included on work limitations that are related to the safe performance of tasks at hand, we feel that finding an additional 10 % of workers could be relevant in an examination that is aimed at verifying whether or not the workers are fit to safely perform their routine and emergency duties.

The high proportion of rail safety workers that perceive difficulties regarding specific activities during their rail safety tasks presents a clear signal. Furthermore, our study indicates that the health-focused approach in fit to work assessments provides too narrow a view on the actual abilities of the worker to meet the requirements of their job. As a result, problems and difficulties that the workers perceive during their daily safety tasks may be overlooked. Consequently, those workers might endanger others or themselves. We believe that including specific work limitations in a fit to work assessment would increase the physician’s understanding of the physical, mental and emotional fitness of the worker. The physician can then make more well-reasoned decisions concerning whether and what type of interventions are needed in order to restore optimal work functioning. In that way, effective interventions (i.e., individual, technical or organizational) aimed at reducing or removing work limitations can be operationalized in a timely and purposeful way.

The assessment of job-specific work limitations has been explored among other occupations and within the scope of preventive strategies, such as workers’ health surveillance programs (Boschman et al. 2014b; Gartner et al. 2012). The specific approach in assessing work limitations has also been explored for patients with specific diseases (Nexo et al. 2015) or demands, such as caregivers (Lerner et al. 2015). The authors acknowledged the potential of specific instruments to provide important insights into relevant limitations.

Any screening strategy will be subject to false positives and false negatives, and our proposed strategy might increase the number of false positives. From a safety perspective, reducing the number of false negatives would be of great concern in our opinion. In the future, it would be worthwhile to assess the positive and negative predictive value of our screening strategy.

A few particular limitations of the present study should be acknowledged. The cross-sectional design limits the scientific robustness of the data. However, for the purpose of this exploratory study—comparing the presence of possible health complaints to current work limitations among a large group of workers—we believe we applied a valid approach.

Supplementing self-perceived work limitations with information based on physical performance tests or observations during work would most likely increase the validity of the assessment of physical work limitations (Boschman et al. 2013). It is likely that some restrictions in task performance might be unnoticed by the workers and tests or observations provide the opportunity to observe those restrictions.

Additionally, a healthy worker effect might have masked the actual relationship between the health problems and work limitations that the older groups of rail safety workers experience (Li and Sung 1999). Older age in itself was not a risk factor for work limitations in general, as we found no statistically significant association between work limitations and the different age groups. Workers in the age group 40–50 years were only at increased risk for having problems or difficulties regarding physical requirements, whereas this was not found for the age group over 50 years. This might be an indication of the healthy worker effect. The associations between workers with health complaints compared to workers with no health complaints and work limitations should therefore be interpreted with some caution. The older workers that experience more health complaints and/or work limitations might have left the occupation, possibly due to a negative judgment of their fitness to work (Zevallos et al. 2014). In a population of workers currently obliged to undergo fit to work assessments based on their age, this should be kept in mind and our conclusions should be interpreted with caution.

The present study is based on self-report by employees and therefore we cannot rule out bias. Another source of bias might be due to selection, as there was no pressure to participate. We have no information about the non-respondents, but based on the distribution among the age categories and the rail safety tasks, we have no reason to believe that our sample is not representative.

Furthermore, we acknowledge that the use of existing validated questionnaires is preferred, instead of using self-developed questions. However, we felt that the assessment of work limitations should be as specific and factual as possible for the rail safety tasks in order to increase the content validity. A priori, we have chosen high content validity as more important than using a general questionnaire. These sources of bias might have resulted in an imprecise estimation of the number of workers with work limitations. Next to this, if symptoms and reported work limitations may affect future employment, this might possibly impact employee honesty. Therefore, it is important to point out to workers that the assessment of the fitness for work is not a goal in itself, but that it is a strategy to timely intervene and restore health, reduce work limitations and facilitate the worker in sustaining their work ability.

The next step would be to compare the effectiveness of assessing work limitations in fit to work assessments, preferably by means of a randomized controlled trial. An intervention study in which safe task performance is the outcome (i.e., whether or not the rail safety worker is able to meet his or her daily job demands) would allow testing of the hypothesis that assessing work limitations is of added value for workers active in jobs that require fit to work assessments. A longitudinal design would enable the effect of fit to work assessments on sustainable employability to be explored (Mahmud et al. 2010).

Based on the findings in our study, we suggest a novel approach for fit to work assessments of rail safety workers that includes an assessment of work limitations in addition to the traditional approach of assessing health complaints and diseases.

## Key points

1. We suggest an improved approach for fit to work assessments for rail safety workers that includes the assessment of task-specific work limitations in addition to the assessment of health complaints.
2. We found statistically significant differences between the proportions of reported health complaints (2–26 %) and work limitations (10–32 %).
3. Workers older than 40 years of age are not at increased risk for work limitations in general, but workers across all age groups with health complaints related to vigilance and clear judgment and physical abilities, are.
